# Late presentation of sorafenib-associated rash: a case report

**DOI:** 10.1186/1752-1947-4-338

**Published:** 2010-10-25

**Authors:** Thomas Sarkodie, Paul Ross

**Affiliations:** 1 Department of Medical Oncology, King's College Hospital, London SE5 9RT, UK

## Abstract

**Introduction:**

Sorafenib, an oral multitargeted tyrosine kinase inhibitor, is licensed for the treatment of hepatocellular carcinoma. Rash is one of the most common side effects of its use, generally appearing within days to a few weeks of commencing treatment. We report the first case of rash appearing nine months after starting treatment with sorafenib.

**Case presentation:**

A 75-year-old Caucasian man initially presented with asymptomatic transient jaundice. He was diagnosed with Barcelona Clinic Liver Cancer stage B hepatocellular carcinoma after extensive investigation. He tolerated sorafenib 400 mg twice a day before presenting nine months later with a rash, confirmed to be drug-induced.

**Conclusions:**

Sorafenib is a drug of choice in Barcelona Clinic Liver Cancer stage B hepatocellular carcinoma. It can cause protracted rash quite late into treatment. Successful management of the rash could contribute to achieving stable disease in hepatocellular carcinoma over a significant period of time.

## Introduction

Hepatocellular carcinoma (HCC) is the fifth most common neoplasm in the world and the third most frequent cause of cancer death worldwide [1]. The incidence of HCC is 500,000 to one million cases per year.

HCC is closely associated with hepatic cirrhosis in the western world, being found in about 70% of all cases. Treatment of HCC is effective in improving survival only when diagnosed at an early stage of the disease.

Tumor staging at diagnosis is essential in deciding appropriate treatment. The most recognized staging systems for HCC currently include the (i) Okuda staging system, (ii) Cancer of the Liver Italian Program (CLIP) scoring system and the (iii) Barcelona Clinic Liver Cancer (BCLC) staging (Tables 1 and 2) [2].

**Table 1:** The BCLC staging

		Tumor Status		
BCLC Stage	PS	Tumor Stage	Okuda Stage	Liver function

Stage A: early				

A1	0	Single, <5 cm	I	No portal HT, normal bilirubin

A2	0	Single, <5 cm	I	Portal HT, normal bilirubin

A3	0	Single, <5 cm	I	Portal HT, normal bilirubin

A4	0	3 tumors, <3 cm	I-II	Child-Pugh A-B

Stage B				

Intermediate HCC	0	Large multinodular	I-II	Child-Pugh A-B

Stage C: advanced	1-2	Vascular invasion/extrahepatic spread	I-II	Child-Pugh A-B

Stage D: end stage	3-4	Any	III	Child-Pugh C

**Table 2:** Okuda Staging System.

		POINTS	
		0	1

Tumor size		<50% liver	>50% liver

Ascites		No	Yes

Albumin (g/dL)		≥3	<3

Bilirubin (mg/dL)		<3	≥3

**Okuda Stage**	**Points**		

I	0		

II	1-2		

III	3-4		

Using the BCLC staging stage A can be managed with radical therapies, such as resection, transplantation or percutaneous treatments. Stages B and C are managed with new agents in clinical trials or palliative care. Transarterial chemoembolization (TACE) is beneficial but is limited by patient suitability and maximum dose of chemotherapy agent used [3,4]. Stage D is for symptomatic treatment [5].

Sorafenib is an oral multitargeted tyrosine kinase inhibitor. It inhibits the receptor tyrosine receptors (RTKs), VEGFR 1-3 (vascular endothelial growth factor receptor), FLT-3 (fms-like tyrosine kinase receptor-3), PDGFR (platelet-derived growth factor receptor ) and the non-receptor serine threonine kinases BRAF (B-Raf proto-oncogene serine/threonine-protein kinase) and (C-Raf proto-oncogene serine/threonine-protein kinase) CRAF. The BRAF and CRAF kinases are members of the RAF/(mitogen-activated protein kinase) MEK/(extracellular-signal-regulated kinases) ERK signaling cascade, which is involved in the survival and proliferation of tumor cells and is a therapeutic target in cancer [3-16].

Phase I trials of sorafenib demonstrated, in addition to safety, promising efficacy in HCC. It was well tolerated; most adverse events were mild to moderate in severity, with most reported side effects at any grade being fatigue (40%), anorexia (35%), diarrhea (34%), rash/desquamation (27%) and hand-foot skin reaction (25%) [4,6,11].

Phase II trials evaluated efficacy and pharmacokinetics of sorafenib in HCC and demonstrated anti-tumor activity in HCC. In one trial of 137 patients, 2.2% of patients achieved a partial response and 33.6% had stable disease over 16 weeks [7]. A subsequent multi-center phase III, double blind, placebo-controlled trial (SHARP trial) involving 602 patients with advanced HCC was conducted. The median survival was 10.7 months in the sorafenib group and 7.9 months in the placebo group (*P *≤ 0.001) [15].

## Case presentation

**Figure 1:**
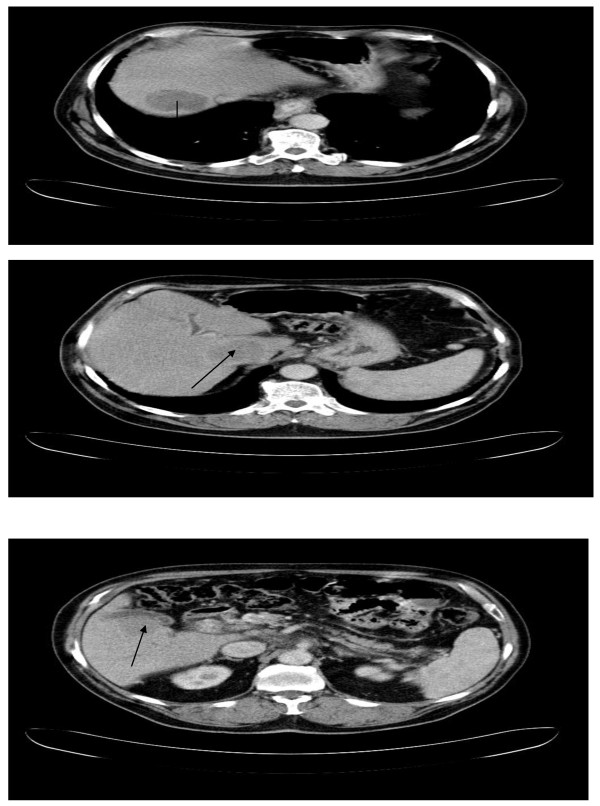
(A-C) CT scans (May 2005): HCC lesions in segment 7, caudate lobe, segment 5 of liver, respectively.

A 75-year-old Caucasian man was referred for consideration of systemic therapy after being diagnosed with HCC. Prior to this diagnosis, he had been fit and well. Past medical history was notable for type 2 diabetes mellitus diagnosed a year earlier, for which he took metformin. He initially presented jaundiced, which cleared spontaneously. Endoscopic retrograde cholangiopancreatogram (ECRP) was normal. Computed tomography (CT) scan (Figure 1A-C) demonstrated three large liver lesions, with the largest measuring under 5 cm in diameter, biopsy of which confirmed HCC.

**Figure 2:**
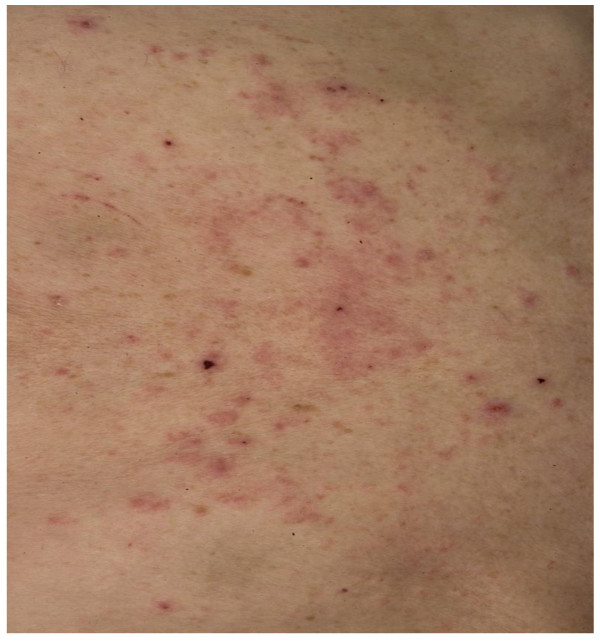
Drug reaction: rash on trunk.

**Figure 3:**
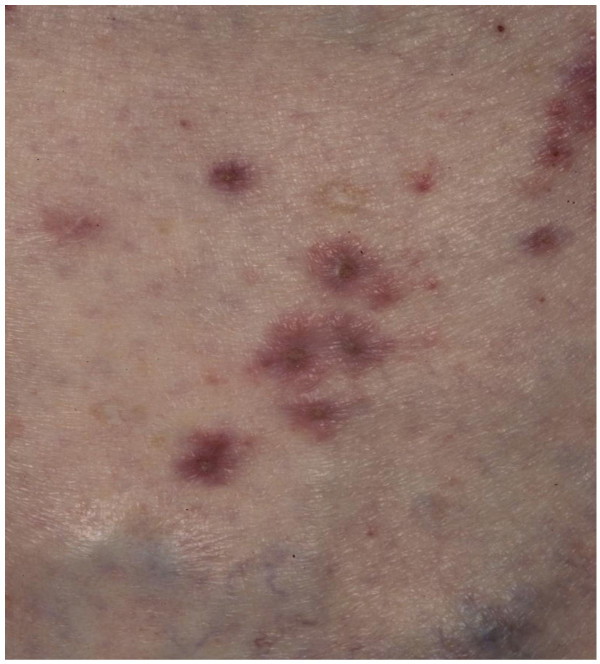
Drug reaction: rash on trunk.

He was well with performance status (PS) 0, and a Child-Pugh score A, AFP (alpha-fetoprotein) 604 ku-L. His BCLC staging was B (Intermediate). A study of sorafenib versus placebo was discussed with our patient and he was entered into the trial in September 2005. He was started on a trial medication at a dose of 400 mg twice a day. The initial side effect experienced was grade I diarrhea managed with loperamide. CT scans a month into treatment demonstrated stable disease. Our patient was reviewed monthly. He remained well with stable disease. Nine months after commencing therapy he presented with a rash. This was initially a dry scaly, itchy rash, showing signs of early lichenification, mainly over the lower half of the abdomen (Figure 2). It gradually spread from the trunk to the arms within six weeks, and became pustular. Topical emollients were commenced with onset of the rash but their effect remained moderate after four months. At this point the rash flared up with some multiple ulcerations over the trunk and arms (Figure 3).

**Figure 4:**
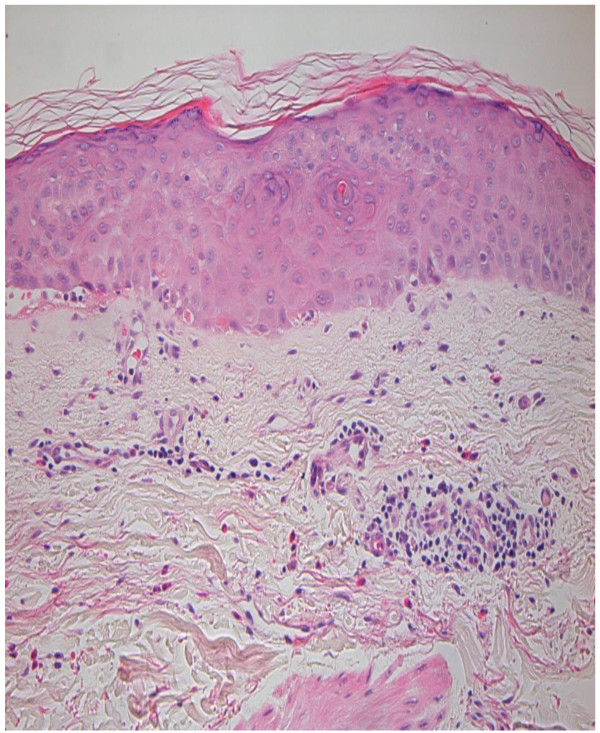
Histology of skin rash: drug reaction.

He was reviewed by a dermatologist and a working diagnosis of eczematous drug reaction was made. Biopsies were taken and the histology was reported as being in keeping with a drug reaction (Figure 4). Our patient was prescribed Fucibet cream (containing betamethasone 0.1%, fucidic acid 2%) to be applied twice daily for a week. The rash improved on subsequent reviews.

Fourteen months after commencing treatment (November 2006) our patient presented with a further rash of common toxicity criteria (CTC) grade 2 over his trunk and limbs. Study medication was stopped at that point. Four weeks later the rash had resolved so the trial medication was recommenced at a reduced dose of 400 mg daily. Our patient remained stable on monthly reviews. Four months later he developed a similar rash as previously; pruritic, mainly on the trunk, but worse on the back. Dermovate (clobetasol propionate 0.05%), diprobase creams and Piriton (chlorphenamine) provided little relief so the trial drug was suspended. Within two months the rash resolved but our patient was not recommenced on trial medication. In spite of that, he again presented a new rash two months later (four months after stopping trial medication). A dermatologist suspected nodular prurigo. Biopsies were taken of the new rash. Histological findings were consistent with nodular prurigo. This was managed with 1% menthol in aqueous cream and 1:4 betnovate ointments and the rash improved to CTC grade 1.

**Figure 5:**
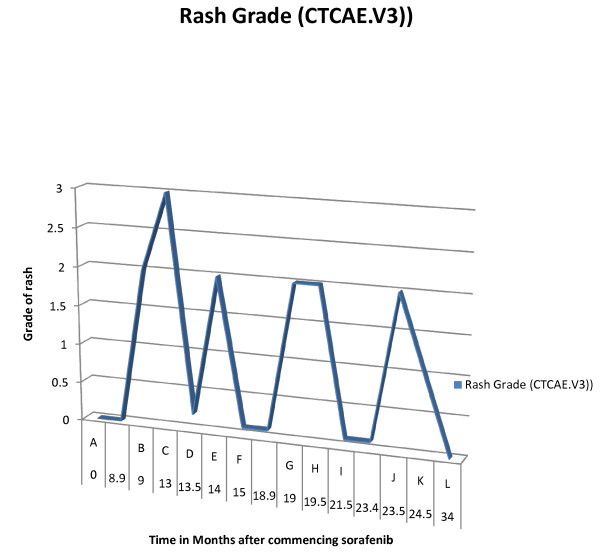
**The course of our patient's rash over time. **(A) Commenced sorafenib 400 mg BiD po. (B) First presentation of rash. (C) Pustular rash with ulceration. (D) Resolution of rash with fucibet cream. (E) Rash flare-up, sorafenib stopped. (F) Rash resolved, sorafenib recommenced at 400 mg qd. (G) Rash recurrence. (H) Sorafenib stopped. (I) Rash resolved, sorafenib NOT recommenced. (J) New rash (nodular prurigo).

**Figure 6:**
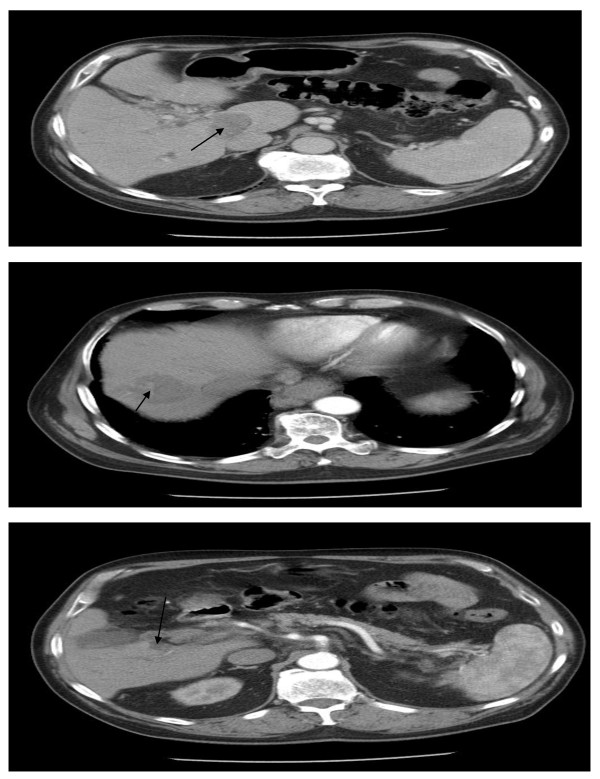
(A-C) CT scans (January 2008): HCC lesions in caudate lobe, segment 7 and segment 5 of liver, respectively.

On the 2^nd ^August 2007 our patient agreed to resume treatment with sorafenib at a dose of 400 mg on alternate days. The rash continued to improve and completely resolved by 30^th ^June 2008 (Figure 5). His latest CT scans at that time (Figure 6A-C) continued to show stable disease, almost three years after commencing treatment.

## Discussion

Sorafenib (Nexavar, Bayer) is a molecular inhibitor of several tyrosine kinases. It targets the Raf/Mek/Erk pathway [3-16]. Its efficacy has been demonstrated in clinical trials [4,6,7,11,16]. Dermatological side effects including rash/desquamation (27%), hand-foot skin reaction (HFSR, 27%), pruritus (18%) and dry skin (10%) are well documented. Other side effects include fatigue (40%), anorexia (35%), diarrhea (40%) hypertension and elevated amylase.

The skin rash usually appears within a few weeks of commencing sorafenib [8-11,17]. The mechanism of the rash is poorly understood. There appears to be a relationship between the eccrine activity of the skin, resulting in higher drug concentration in the areas of the rash or desquamation [8]. Inhibition of HER-1/EGFR in the skin is also thought to play a role in the development of the rash [10]. Some studies found a relationship between the Child-Pugh score prior to commencement of medication and the severity of the adverse drug reaction [4].

The late presentation of the rash in our patient appears to be the first reported of its kind in the literature to date. It first appeared nine months into treatment; it was mild to moderate in severity but was protracted and recurring. Management included moisturizing creams and topical steroids, the latter being of limited use [10]. Further improvements in our understanding of the pathogenesis underlying the side effects are needed in the management of these patients.

**Figure 7:**
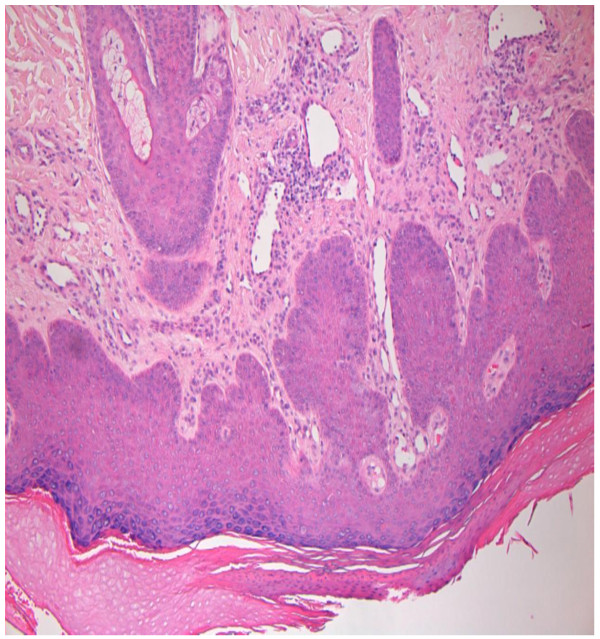
Histology of skin rash: nodular prurigo.

Unanswered questions regarding the protracted nature of the rash in our patient include whether the relatively long period the drug was tolerated prior to onset of the rash, might have contributed to its persistence four months after stopping the drug. Nodular prurigo, which could arise as a result of long-term scratching of the skin to any itchy stimulus, was confirmed in our patient by the second biopsy (Figure 7) as a consequence of the persistence of the original itchy rash. Reintroduction of the drug at a reduced dose after resolution of the rash did not precipitate recurrence. Our patient remained stable at this dose of medication by July 2008.

## Conclusions

Sorafenib, a multitargeted tyrosine kinase inhibitor, is a drug of choice in patients with BCLC stage B HCC. Skin rash is a common adverse drug reaction, occurring within weeks of commencement of the drug. It is, however, important to note that the rash can present quite late into treatment. Prompt recognition and management of the rash remains vital to patients' outcome.

## Consent

Written informed consent was obtained from the patient for publication of this case report and any accompanying images. A copy of the written consent is available for review by the Editor-in-Chief of this journal.

## Competing interests

The authors declare that they have no competing interests.
